# Vulvar Vestibulitis—A Complex Clinical Entity

**DOI:** 10.1155/S106474499600052X

**Published:** 1996

**Authors:** William J. Ledger, Alan Kessler, Garrick H. Leonard, Steven S. Witkin

**Affiliations:** Department of Obstetrics and Gynecology Division of Infection and Immunology New York Hosptial-Cornell University Medical Center New York NY 10021 USA

## Abstract

*Objective:* This study aims to determine the pathophysiology of vulvar vestibulitis and to evaluate currently used treatment options.

*Methods:* Two hundred twenty women with vulvar vestibulitis were seen between October 1987 and March 1995. Every patient had vulvar pain when they attempted intercourse, 75% had excessive vaginal discharge, 36.4% had constant or recurring vulvar burning, and 10.9% had symptoms suggestive of cystitis. All were cultured for the presence of *Candida albicans*. One hundred sixty-one (73.2%) were also tested for vaginal IgE and prostaglandin E_2_ (PGE_2_); 72 (32.7%) had a vulvar biopsy performed as well.

*Results:* A wide range of variants were noted: 53 (24.1%) had a human papilloma virus (HPV) infection, 25 (11.4%) had a Candida vulvovaginitis, 43 (19.5%) had a vaginal allergy, 15 (6.8%) had vaginal PGE_2_ present, 14 (6.4%) had elevated urinary oxalate excretion, and 29 (13.2%) had a variety of diagnosed variants. In 81 (36.8%) no underlying diagnosis was made. This understates the numbers and varieties of vulvar vaginal diagnoses, for not all patients received a vaginal fluid analysis, a vulvar biopsy, or a 24 h urine screen for oxalates. A variety of medical and operative interventions was used. Symptoms were relieved in 65.9% of patients. The degree of sueeess varied. Successful outcomes were achieved in 14.3% of patients using a low oxalate diet and calcium citrate supplementation, 16% with anti-Candida treatment, 48.1% with antihistamines, 77% with vulvar injection of interferon, 83% with operative removal of inflamed vulvar tissue, and a posterior colporrhaphy used to cover the cutaneous defect.

*Conclusions:* The diagnosis of vulvar vestibulitis is easy to make. An etiology for this chronic condition will not be achieved in every patient. A majority of patients can get relief by a variety of medical and operative interventions.


Infectious Diseases in Obstetrics and Gynecology 4:269–275 (1996)
© 1997 Wiley-Liss, Inc.

### Vulvar Vestibulitis—A Complex Clinical Entity

**William J. Ledger,* Alan Kessler, Garrick H. Leonard, and Steven S. Witkin**

*Department of Obstetrics and Gynecology, Division of Infection and Immunology, New York Hospital-Cornell University Medical Center, New York, New York*

ABSTRACT

*Objective:* This study aims to determine the pathophysiology of vulvar vestibulitis and to evaluate currently used treatment options.

*Methods:* Two hundred twenty women with vulvar vestibulitis were seen between October 1987 and March 1995. Every patient had vulvar pain when they attempted intercourse, 75% had excessive vaginal discharge, 36.4% had constant or recurring vulvar burning, and 10.9% had symptoms suggestive of cystitis. All were cultured for the presence of *Candida albicans.* One hundred sixty-one (73.2%) were also tested for vaginal IgE and prostaglandin $E_2$ ($PGE_2$); 72 (32.7%) had a vulvar biopsy performed as well.

*Results:* A wide range of variants were noted: 53 (24.1%) had a human papilloma virus (HPV) infection, 25 (11.4%) had a Candida vulvovaginitis, 43 (19.5%) had a vaginal allergy, 15 (6.8%) had vaginal $PGE_2$ present, 14 (6.4%) had elevated urinary oxalate excretion, and 29 (13.2%) had a variety of diagnosed variants. In 81 (36.8%) no underlying diagnosis was made. This understates the numbers and varieties of vulvar vaginal diagnoses, for not all patients received a vaginal fluid analysis, a vulvar biopsy, or a 24 h urine screen for oxalates. A variety of medical and operative interventions was used. Symptoms were relieved in 65.9% of patients. The degree of success varied. Successful outcomes were achieved in 14.3% of patients using a low oxalate diet and calcium citrate supplementation, 16% with anti-Candida treatment, 48.1% with antihistamines, 77% with vulvar injection of interferon, 83% with operative removal of inflamed vulvar tissue, and a posterior colporrhaphy used to cover the cutaneous defect.

*Conclusions:* The diagnosis of vulvar vestibulitis is easy to make. An etiology for this chronic condition will not be achieved in every patient. A majority of patients can get relief by a variety of medical and operative interventions. Infect. Dis. Obstet. Gynecol. 4:269–275, 1996.
© 1997 Wiley-Liss, Inc.

KEY WORDS

vulvar vestibulitis; HPV; *Candida albicans;* vaginal IgE; $PGE_2$

Physicians should be cognizant of the syndrome of vulvar vestibulitis. Adult women with this painful, socially debilitating malady need emotional support and appropriate medical care. Unfortunately, because of the lack of awareness of this condition, these positive interventions are often not offered. Specific diagnostic techniques must be carried out by the physicians to make the diagnosis, and the majority of patients can be helped by physician treatment. This requires physician patience to deal with a chronic medical problem. It is the purpose of this paper to review our experience with vulvar vestibulitis in the Department of Obstetrics and Gynecology at the New York Hospital-Cornell University Medical Center.

### MATERIALS AND METHODS

The 220 women in this report were all seen in consultation in the time interval from October 1987 until March 1995. Only 4 patients were seen in 1987 and 1988, and then the numbers of consultations increased during the last 6 years surveyed. All patients had a vaginal pH determination and a microscopic examination of the vaginal secretions using saline and potassium hydroxide solution. In 161

*Correspondence to: Dr. William J. Ledger, Department of Obstetrics and Gynecology, Division of Infection and Immunology, New York Hosptial-Cornell University Medical Center, New York, NY 10021.

Received 16 October 1996
Accepted 15 November 1996

TABLE 1. Symptoms

| | |
|---|---|
| Pain | 220 (100%) |
| Excessive vaginal discharge | 165 (75%) |
| Vulvar burning | 80 (36.4%) |
| Urinary tract symptoms | 24 (10.9%) |

TABLE 2. Abnormalities discovered

| | | |
|---|---|---|
| HPV | 53 | (24.1%) |
| Allergic vaginitis (elevated IgE) | 43 | (19.5%) |
| Candida vaginitis | 25 | (11.4%) |
| Elevated $PGE_2$ | 15 | (6.8%) |
| Elevated urinary oxalates | 14 | (6.4%) |
| Miscellaneous | 29 | (13.2%) |
| Desquamative vaginitis | 12 | (5.5%) |
| Micropapillomatosis | 9 | (4.1%) |
| Herpes | 4 | (1.8%) |
| *Chlamydia trachomatis* | 2 | (0.9%) |
| *Neisseria gonorrhoeae* | 1 | (0.45%) |
| Bacterial vaginosis | 1 | (0.45%) |
| Idiopathic | 81 | (36.8%) |

(73.2%) of the patients, a fluid sample was collected for Candida culture and determination of IgE and prostaglandin $E_2$ ($PGE_2$) levels. This testing was not done in some women who had no vaginal symptoms and it was declined by some patients due to cost considerations usually because of membership in a Health Maintenance Organization or some other insurance payment plan that would not reimburse the patient for this test. All of the women who did not have vaginal fluid testing had a culture performed for the presence of yeast and bacteria. Seventy-two patients (32.7%) with a vulvar area that turned white after the application of 4% acetic acid were biopsied and those with microscopic evidence of active human papilloma virus (HPV) infection had a colposcopic examination performed.

### RESULTS

These patients ranged in age from 19 to 63 years, with a median age of 32 years. All had a history of dyspareunia so intense that it eliminated the possibility of sexual intercourse. All had severe point tenderness when a cotton-tipped applicator was applied to small reddened vestibular gland sites at specific anatomic locations. These women had a wide variety of other symptoms (Table 1): 165 (75%) complained of a persistent vaginal discharge, 80 (36.4%) had constant or recurring vulvar burning, and 24 (10.9%) had repeated urinary tract symptoms suggestive of a cystitis, but had no significant bacterial growth on urine culture.

During their diagnostic evaluations, there was a wide variety of vulvovaginal findings. These findings are noted in Table 2. HPV was the most common infection found in this population of women: 53 (24.1%) had a history or present evidence on vulvar biopsy of HPV infection. Thirty-five (15.9%) had a history of HPV infection treated by a variety of local agents, including trichloroacetic acid (TCA), 5-fluorouracil (5-FU), or local laser. In 12 of these patients vulvar pain began after treatment, following laser in 8 cases, 5-FU in 3 cases, and TCA in 1 case. Of the 18 cases with active HPV infection, 13 were treated with local interferon injections. A positive culture for *Candida albicans* was found in 25 women (11.4%). Twenty-four of these 25 women (96%) complained of an excessive vaginal discharge. Many patients without Candida also had a discharge, 145 of 195 (72.3%). These differences are statistically significant. Chi-square using the Yates correction is 5.43 ($P < 0.05$). Despite this, only 24 of the 165 women (14.5%) with an abnormal discharge were culture positive for Candida. Candida was detected on microscopic examination in only 13 of these 15 culture positive cases (52%). Candida forms were also seen on microscopic examination in 2 women who were culture negative. Vaginal IgE, indicating a local vaginal allergy,[1] was found in 43 of the 161 women (26.7%) tested. These 43 women represent 19.5% of the total of the 220 patients who were evaluated. Vaginal $PGE_2$ as the only positive finding was detected in 15 of the 160 screened (9.4%), or 6.8% of the total patient population. Fifteen women who had failed treatment with hydroxyzine or antihistamines had a 24 h urine analysis done by Dr. Solomon to evaluate oxalate excretion.[2] Fourteen (93.3%) of these tests were positive, or 6.4% of the total population. Another 29 (13.2%) had a variety of diagnosed variants: 12 (5.5%) had desquamative vaginitis, 9 (4.1%) had micropapillomatosis labialis, 4 (1.8%) had recurring genital herpes, 2 (0.9%) had *Chlamydia trachomatis*, 1 (0.45%) had *Neisseria gonorrhoeae*, and 1 (0.45%) had bacterial vaginosis. In 81 women (36.8%), no underlying diagnosis was made. The total in Table 2—260—is related to the fact that some women had more than one abnor-

TABLE 3. Treatment outcomes[a]

| | | |
|---|---|---|
| Antihistamines | 102 of 212 | (48.1%) |
| Operations | 25 of 30 | (83%) |
| Interferon | 10 of 13 | (77%) |
| Anti-Candida | 4 of 25 | (16%) |
| Low oxalate diet and citrate ingestion | 2 of 14 | (14.3%) |
| Other | 2 | |

[a]Improved: 145 of 220 (65.9%).

mality noted. Table 2 obviously understates the numbers and varieties of vulvovaginal pathology for all patients who did not receive a vaginal fluid analysis, a vulvar biopsy, or a 24 h urine screen for oxalates. For example, a recent polymerase chain reaction (PCR) screen for HPV found it present in 54% of 46 operative samples of women with vulvar vestibulitis.[2] Prior to our seeing these patients, all had been evaluated for the presence of *N. gonorrhoeae* and most had been screened for *C. trachomatis*. At the time of our evaluation, we did not do a PCR for *C. trachomatis* or *Trichomonas vaginalis* and no serum antibody testing was done for herpes simplex virus-2 (HSV-2).

Any analysis of treatment requires a definition of physician assessment of patient response to therapy. Few women were totally cured of the symptoms noted in Table 1. After treatment deemed successful by this group of investigators all patients were not completely free of pain, vaginal discharge, vulvar burning, or urinary tract symptoms. The satisfactory treatment response noted in Table 3 indicates an improvement in patient symptomatology to the point that they were able to have intercourse again. We also observed some patients classified as a cure who had a recurrence of pain, particularly in the postpartum period. This symptom flare-up responded to medical treatment.

The treatment outcomes are noted in Table 3. Only 65.9% were cured after a variety of treatments. Some explanations are necessary for the analysis of these results. All but 8 of the 220 women received either hydroxyzine or an antihistamine when they were first seen. In those patients who failed to respond to this initial therapy, and whose vaginal secretions and cultures showed no evidence of a specific etiology, a biopsy or alternative therapy was used. A urine screen for oxalate excretion was reserved for treatment failures who had persistent vulvar burning, while an operation was performed in treatment failure patients who continued to have severe pain when they attempted intercourse. This accounts for the small numbers in those treatment categories.

The treatment results were less promising than expected (Table 3). Less than half, 102 of 212 (48.1%), of the women treated with hydroxyzine or an antihistamine responded to therapy. Almost all patients had some improvement in symptoms, but the majority did not have enough pain relief to resume having intercourse. This poor response was even true in the 43 women who had allergic vaginitis confirmed by the presence of an elevated IgE in vaginal secretions; 42 or 43 were treated, with 21 cured (50%). Of the 118 tested women without IgE present, 110 were treated with antihistamines and 53 were cured (48.2%). These differences are not statistically significant. Thirty of the 220 women who failed to respond to medical treatment had an operation with removal of the vestibular glands and the inflamed vulvar tissue. A posterior colporrhaphy was done to mobilize the posterior wall of the vagina and this was used as a graft to cover the cutaneous defect caused by the operation. Twenty-five of 30 women (83%) were improved by this approach, although 2 of these women required 2 operations to get a satisfactory result. In 13 women with a symptomatic vulvar HPV infection, 12 separate interferon injections, each one million units of alpha 2B, were given subcutaneously in the vulva over a 4 week interval. Ten (77%) had a satisfactory response with relief of pain 6–8 weeks after therapy and no gross posttreatment changes in the vulvar epithelium were noted. Other treatment regimens had a much lower success rate. Twenty-three of the 25 women (92%) had Candida eliminated from the vagina on follow-up culture after oral anti-Candida treatment. However, only 4 (16%) had relief of symptoms. Fourteen of 15 women who submitted 24 h urine samples for oxalate evaluations had elevated levels, but only 2 of the 14 (14.3%) were able to tolerate the low oxalate diet and the recommended high calcium citrate regimen for the long periods of time that were required.[3] Despite this discouraging treatment picture, there are glimmers of hope. In this study population of 220 women, 20 (9.1%) became pregnant and successfully carried their pregnancies to term. In addition, no serious underlying diseases were found in this population of women during this time interval of observation.

### DISCUSSION

Physician awareness of this syndrome, vulvar vestibulitis, is important. Too many obstetrician-gynecologists fail to diagnose this syndrome. For the symptomatic patient, this translates into a series of visits to many different doctors in which the recurring physician response is a lack of recognition of the problem. Since the busy doctors, overburdened with large numbers of patients with easily recognized conditions, such as pregnancy, do not see any gross abnormalities, they dismiss the patient. These women leave with a new layer of iatrogenic concern; since their physicians see no pathology, perhaps their symptoms are not real.

The physician should have no difficulty in making the diagnosis of vulvar vestibulitis. A directed history will yield crucial facts. All of these women have introital pain when they try to have intercourse. For most patients, their vulvar discomfort is only related to attempts to have intercourse, but in this study population, over a third of the women had constant vulvar burning (see Table 1). The majority of the 220 women (75%) have an excessive vaginal discharge and a minority (10.9%) had chronic urinary tract symptomatology. The number of patients with lower urinary tract symptoms (10.9%) is lower than the 44% reported by McCormack.[4] After obtaining a history, the diagnosis can be confirmed at the time of physical examination. Pressure with a cotton-tipped applicator stick at specific pressure points (picture the vulva as the face of a clock) at 3:30 and 8:30 elicits excruciating pain. This physical diagnosis finding of pain at a specific anatomic site whose small vestibular gland openings are easily identified is the reason we favor the terminology vestibular adenitis for this syndrome. It is a reminder of an important physical diagnosis abnormality. Other authors[4,5] have used the designation focal vulvitis; they object to the term vestibular adenitis for they do not find inflamed vestibular glands in the pathology specimens of these patients. We do not dispute this for in our review of the pathologic specimens in this series, this was the exception rather than the rule. In contrast, a histopathologic study of 41 cases of vulvar vestibulitis found vestibular glands present in 66% of the cases.[6] The frequency of involvement of the vestibular glands in operative specimens may be related to the number of microscopic sections obtained. We acknowledge the controversy about naming this syndrome and accept the current designation vulvar vestibulitis.[6] Whatever terminology is used, the diagnosis can be confirmed by the physician within minutes at the time of the initial examination with the use of a cotton-tipped applicator stick.

Although there is no common infectious agent in this syndrome, clinical observations suggest a uniform response in the progression and maintenance of the pain of vestibular adenitis. Most of these women have the onset of symptomatology associated with a specific local inflammatory event. The triggering factor can vary. This can be inflammatory vulvitis due to an HPV infection, vulvovaginitis due to *C. albicans*, an allergic vulvovaginitis, or inflammation resulting from the local treatment of "flat warts," particularly by laser. What distinguishes these patients from most women with an inflammatory vulvovaginitis is the persistence of the inflammation and pain.

Some physicians have likened the persistent vulvar pain in this syndrome to the sympathetic dystrophy syndrome.[7] They theorize that cutaneous vulvar disturbances destabilize the pelvic floor muscles. This destabilization of the pelvic muscles perpetuates vulvar tissue inflammation by its effects on locally autonomic (sympathetic) mediated activity. This results in vulvar vascular changes and histamine release. This theory parallels the clinical observations in these patients and is the justification for many of the medications used. Hydroxyzine reduces sympathetic nerve activity and relaxes pelvic muscles. Antihistamines block the effects of the local release of histamines. Laser in the rapid super pulse mode has been used to eliminate excessive vascularization.[8] Biofeedback has been employed to correct pelvic floor disability.[7] All of these regimens help some but not all patients. Some studies have suggested a psychologic component to this syndrome and suggest that stress plays a role in dyspareunia and vulvar pain.[9,10] One study demonstrated improved results when psychologic treatment was included in the regimen.[9] These theories are a search for an explanation for the chronic nature of this problem.

Currently available therapeutic strategies for these patients can be frustrating for both the patient and the physician. These women have a chronic vulvar inflammatory problem that usually requires long periods of time, i.e., months, to achieve a satisfactory response. In addition, successful therapy often necessitates more than one type of therapeutic intervention to achieve a cure. Besides the focus on the causes of the local vulvar inflammation, the physician has to be cognizant of the pelvic floor muscle destabilization and sympathetic nerve hyperactivity which perpetuate the vulvar inflammation and pain. A variety of approaches have been utilized to minimize these responses.

Therapy begins with a search for an etiology for the continued vulvar inflammation. If this is found, specific medications can be prescribed. The patient response rate varies with each determined source of inflammation. The treatment results shed some light on the pathophysiology of this chronic condition.

Our outcomes in patients with Candida infections provide insights for therapy strategy. Candida vulvovaginitis was documented by a positive culture in 25 women (11.4%) of the total. They had expected symptomatology. Twenty-four of 25 women (96%) of this subpopulation complained of an excessive vaginal discharge. Although an excessive vaginal discharge was more common in women culture positive for Candida (96% vs. 71.8%), physicians caring for women with vulvar vestibulitis must be cautious in attributing the excessive vaginal discharge to a Candida infection. There are two reasons for this caution. Candida is an uncommon cause of this symptomatology. Only 24 of the 165 women (14.5%) with an abnormal vaginal discharge were culture positive for Candida vaginitis. In addition, Candida vaginitis is a difficult diagnosis to make in this population. Only 13 of the 25 culture positive women (52%) had yeast forms detected in the wet mount microscopic examination of vaginal secretions that were Candida culture positive. These results are not unexpected for they are similar to those reported by McCormack et al.[11] in their evaluation of women with a symptomatic yeast vaginitis. In women with vulvar vestibulitis, diagnosis of a yeast infection requires culture confirmation. Beside diagnostic difficulties, there are treatment problems. Our treatment results show that Candida infections were not an important factor in maintaining the vulvar pain suffered by these women. The anti-Candida oral treatment was effective in eliminating Candida, but it relieved symptoms in only 4 of 25 (16%). The failure of patient symptom response to antifungal treatment should not be a signal for continued long-term antifungal treatment. In this population, these women remained symptomatic despite a microbiologic cure. Continued antifungal treatment for a culture negative patient has no rationale and increases the possibility of an adverse reaction to antifungal medications, particularly local forms such as creams and suppositories containing propylene glycol.

Our results with attempts to decrease urinary oxalate secretion were just as disappointing as the antifungal treatment. Only 2 of 14 (14.3%) had an improvement in symptoms. It is difficult for us to gauge the significance of excessive oxalate excretion in this population. Based upon laboratory evaluations done by Dr. Solomon's laboratory, it was a common problem. Fourteen of the 15 women surveyed (93.3%) had excessive oxalate excretion. Although commonly found when tests were done, the treatment results were poor. Only 2 of the 14 (14.3%) had an improvement in symptoms with a low oxalate diet and heavy calcium citrate ingestion. Although these patients were counseled before treatment that up to 6 months of therapy would be necessary for a cure, the lack of improvement and the diminished quality of life with the diet and abdominal bloating with the citrate caused 12 of the 14 to stop treatment.

In contrast, the immunologic treatment of patients with an active HPV infection was associated with a higher incidence of success. Ten of 13 such women (77%) treated with local interferon injections were cured. This is a long course of treatment. The acute treatment phase, 12 injections given 3 times/week, requires 4 weeks for completion. The posttreatment recovery phase is 8 weeks before a judgment can be made about success or failure of treatment. Despite this length of time, the therapeutic response was gratifying. There was a high cure rate and the treatment failures had no vulvar scarring with which to contend.

The physician caring for women with vulvar vestibulitis should be alert to the possibility of local allergy. Forty-three women (19.5%) of the total had evidence of a local vaginal allergy. The treatment results were disappointing. All but 1 of these patients were treated with antihistamine for several months and only 21 patients (50%) were improved. A major diagnostic and therapeutic problem in women with an allergic vaginitis is that the vaginal IgE is not present in the serum.[1] This eliminates the possibility of skin testing for specific antigens so that the patient can avoid future exposure. This would be a preferable option if it were available. The high frequency of vaginal allergy in women with vulvar vestibulitis should be acknowledged by physicians when vaginal IgE testing is not available. Many of these women are sensitive to local creams and suppositories which contain propylene glycol. These local agents should be avoided.

The results of this study indicate that treatment strategy must look beyond immediate causes of vulvar irritation. There are a number of observations that support this view. When a specific trigger for the irritation was determined, not all of the patients were cured. There was a wide range of success from 14.3% in those with excessive oxalate excretion to 77% in those with an active HPV infection treated with interferon (see Table 3). In the case of Candida, a microbiologic cure did not equate with clinical success. In the others, the failure in part could be related to shortcomings of the therapy. In addition to these treatment failures, there were 81 women (36%) of the total in whom no discernible cause of the vulvar irritation was discovered. These numbers might have been lower if a vulvar biopsy had been performed in every patient.[2] If therapy is focused solely upon obvious causation of the vulvar irritation, a large segment of the patient population has no hope for relief.

Long-term treatment is needed to achieve a clinical cure. The best explanation for the results seen is that autonomic nerve dysfunction results in continuing vulvar irritation and destabilization of the pelvic muscles. Success of antihistamines or hydroxyzine was only noted after months of therapy. It was found in 21 of 42 (50%) of the patients with a demonstrable vaginal allergy. This therapeutic intervention was almost as successful when used in those tested women without a vaginal allergy. Fifty-three of 110 tested women (48.2%) who were treated responded. The response of these patients to antihistamines when no local allergy was detected reinforces the hypothesis that neurologic disturbances (i.e., autonomic nerve dysfunction) may also trigger histamine release.

The low rate of success with hydroxyzine and antihistamines has spurred an interest in alternate therapeutic approaches. Biofeedback methods have been noted to have success in some patients with this chronic vulvar problem.[7] We believe our high success rate with operative intervention (83%) is related to postoperative therapy that addresses the problem of pelvic muscle destabilization. After the vulvar sutures have reabsorbed, we place these patients on a program of progressive introital dilatation with vaginal dilators. These are used in progressively larger sizes over a 6 week period, before intercourse is attempted. These women discover with this progressive program that the vagina can accept objects the size of an erect male penis without pain. This process of patient controlled vaginal dilatation has been a major factor in our high operative success rate, but a recent study by Goetsch[12] had excellent results without this postoperative care. Other factors are important in any assessment of operative intervention. In this series, operations were the last therapeutic resort for patients who were medical treatment failures, whose problem was pain without constant vulvar burning. We believe this is important. A rush to operation as initial treatment will yield failures and the subsequent management of these women is difficult. Operation should not be the first line of care.

### REFERENCES

1. Witkin SS, Jeremias J, Ledger WJ: A localized vaginal allergic response in women with recurrent vaginitis. J Allergy Clin Immunol 81:412–416, 1988.
2. Bornstein J, Shapiro S, Rahat M, Goldschmid N, Goldik Z, Abramovici H, Labat N: Polymerase chain reaction search for viral etiology of vulvar vestibulitis syndrome. Am J Obstet Gynecol 175:139–144, 1996.
3. Solomons CC, Melmed MH, Heitter SM: Calcium citrate for vulvar vestibulitis: A case report. J Reprod Med 36:879–882, 1991.
4. McCormack WM: Two urogenital sinus syndromes. Interstitial cystitis and focal vulvitis. J Reprod Med 35: 873–876, 1990.
5. Peckham BM, Maki DG, Patterson JJ, Hafez GR: Focal vulvitis: A characteristic syndrome and cause of dyspareunia: Features, natural history, and management. Am J Obstet Gynecol 154:855–864, 1986.
6. Pyke RE, Wilkinson EJ, Friedrich EG Jr, Croker BP: The histopathology of vulvar vestibulitis syndrome. Int J Gynecol Pathol 7:249–257, 1988.
7. Glazer HI, Rodke G, Swencionis C, Hertz R, Young AW: Treatment of vulvar vestibulitis syndrome with electromyographic biofeedback of pelvic floor musculature. J Reprod Med 40:283–290, 1995.

8. Reid R, Greenberg MD, Drood Y, Husain M, Seluggi S, Wilkinson E: Colposcopic findings in women with vulvar pain syndromes. A preliminary report. J Reprod Med 33:523–532, 1988.
9. Schorer LR, Youngs DD, Cannata R: Psychosexual aspects of the evaluation and management of vulvar vestibulitis. Am J Obstet Gynecol 167:630–636, 1992.
10. Lynch PJ: Vulvodynia: A syndrome of unexplained vulvar pain, psychologic disability, and sexual dysfunction. J Reprod Med 31:773–780, 1986.
11. McCormack WM, Starke KM, Zinner SH: Symptoms associated with vaginal colonization with yeast. Am J Obstet Gynecol 158:31–33, 1988.
12. Goetsch MF: Simplified surgical revision of the vulvar vestibule for vulvar vestibulitis. Am J Obstet Gynecol 174:1701–1707, 1996.
